# Effects of physical activity in women with polycystic ovary syndrome: a systematic review and meta-analysis

**DOI:** 10.61622/rbgo/2025rbgo56

**Published:** 2025-09-12

**Authors:** Diana Carvalho Braga Cavalcante, Thalita Basso Scandolara, Gislaine Satyko Kogure, Cainã Rodrigues, Carolina Gennari Verruma, Manoel Odorico de Moraes, Rosana Maria dos Reis, Marcelo Borges Cavalcante, Cristiana Libardi Miranda Furtado

**Affiliations:** 1 Universidade Federal do Ceará Fortaleza CE Brazil Universidade Federal do Ceará, Fortaleza, CE, Brazil.; 2 Faculdade de Medicina de Ribeirão Preto Universidade de São Paulo Ribeirão Preto SP Brazil Faculdade de Medicina de Ribeirão Preto, Universidade de São Paulo, Ribeirão Preto, SP, Brazil.; 3 Faculdade de Medicina Universidade Federal do Ceará Fortaleza CE Brazil Faculdade de Medicina, Universidade Federal do Ceará, Fortaleza, CE, Brazil.; 4 Faculdade de Medicina Universidade de Fortaleza Fortaleza CE Brazil Faculdade de Medicina, Universidade de Fortaleza, Fortaleza, CE, Brazil.; 5 Universidade Federal de Minas Gerais Belo Horizonte MG Brazil Universidade Federal de Minas Gerais, Belo Horizonte, MG, Brazil.

**Keywords:** Physical exercise, Polycystic ovary syndrome, Resistance training, Meta-analysis

## Abstract

**Objective:**

To evaluate the effects of aerobic and resistance exercise on body mass index (BMI), insulin levels, lipid profiles, and hormonal parameters in women with polycystic ovary syndrome (PCOS).

**Data sources:**

We searched PubMed, Web of Science, and Embase databases for publications up to September 2024. Fully published articles involving reproductive-age women diagnosed with PCOS were included.

**Study selection:**

Randomized controlled trials comparing supervised aerobic or resistance exercise to no intervention in women diagnosed with PCOS based on Rotterdam or NIH criteria were included. The Rayyan Systematic Review tool was used to organize study data.

**Data collection:**

Data extraction was conducted independently by two reviewers. Meta-analysis employed random-effects modeling.

**Data synthesis:**

Ten randomized controlled trials with 382 women were analyzed. Aerobic exercise interventions (12–24 weeks) reduced BMI, waist circumference, insulin levels, total cholesterol, and low-density lipoprotein. Effects on fasting glucose, high-density lipoprotein, and triglycerides varied. Hormonal assessments showed reduced testosterone and increased sex hormone-binding globulin in some studies. Resistance exercise improved lean body mass and reduced body fat percentage but showed minimal effects on hormonal parameters. Meta-analysis revealed aerobic exercise decreased insulin, cholesterol, and triglycerides compared to no intervention, while other metabolic and hormonal markers showed inconsistent changes.

**Conclusion:**

Aerobic and resistance exercise improve anthropometric measures, metabolic health, and hormonal balance in women with PCOS. These findings highlight exercise as a valuable therapeutic strategy for managing PCOS and enhancing overall health outcomes.

## Introduction

Polycystic ovary syndrome (PCOS) is a complex disorder affecting 5-20% of women of reproductive age, characterized by metabolic, hormonal, and psychological changes that lead to chronic anovulation and impaired reproductive function.^(1,2)^ Clinically, PCOS manifests as irregular menstrual cycles, ovarian cysts, and hyperandrogenism.^(3)^ Androgen excess is a key driver of infertility and metabolic dysfunction in PCOS, which also elevates the risk of cardiovascular disease (CVD) and type 2 diabetes mellitus (T2DM).^(2)^

Although the physiopathology of PCOS remains unclear due to its heterogeneous phenotypes and comorbidities, it is widely considered a multifactorial disorder with genetic, epigenetic, and environmental influences.^(4)^ Factors such as low birth weight, early puberty, weight gain, and sedentary lifestyles increase susceptibility.^(5-10)^ Obesity or overweight, which occurs in 38–88% of women with PCOS depending on the population, exacerbates insulin resistance (IR) and hyperandrogenism, creating a cycle of metabolic disturbances and anovulation.^(11)^ Notably, obesity serves as both a risk factor and trigger for PCOS during puberty.^(12)^

The 2023 International Evidence-Based Guideline for the Evaluation and Treatment of PCOS highlights lifestyle changes as the primary treatment, emphasizing dietary modifications and regular exercise.^(3)^ Aerobic exercise improves body composition, insulin sensitivity, and lipid profiles, while strength training reduces body fat, waist circumference, and hormonal imbalances.^(13-16)^ Thus, the effects of physical activity on PCOS outcomes may vary depending on the type and duration of exercise.

Physical exercise is a promising strategy to address the metabolic and hormonal imbalances in PCOS, improving overall quality of life and reproductive health. However, the benefits depend on the type, intensity, and duration of exercise. This systematic review and meta-analysis aim to synthesize evidence on the effects of aerobic and resistance exercise on body mass index (BMI), insulin levels, lipid profiles, and hormonal parameters in women with PCOS, providing insights for more effective and tailored treatment strategies.

## Methods

This systematic review and meta-analysis followed the PRISMA guidelines and was registered in the International Prospective Register of Systematic Reviews (PROSPERO: CRD42024474048). It included randomized controlled trials (RCTs) that evaluated the impact of aerobic or resistance exercise in women with PCOS. The diagnostic criteria were based on either the Rotterdam consensus, which requires at least two of the following features: oligomenorrhea and/or chronic anovulation, clinical and/or laboratory signs of hyperandrogenism, or polycystic ovaries identified through ultrasound;^(17,18)^ or the NIH criteria, which require both clinical and/or biochemical evidence of hyperandrogenism along with ovulatory dysfunction while excluding other possible causes.^(19)^

A systematic search was conducted in the PubMed, Web of Science, and Embase databases for literature published up to September 2024, with the search completed in December 2024. Different MeSH terms were used for the search strategies on all databases: PCOS AND (“resistance exercise” OR “resistance training” OR “aerobic exercise” OR “aerobic training” OR “exercise”). Mesh terms (Medical Subject Headings): (“Polycystic Ovary Syndrome/classification”[Majr] OR “Polycystic Ovary Syndrome/complications”[Majr] OR “Polycystic Ovary Syndrome/diagnosis”[Majr] OR “Polycystic Ovary Syndrome/diagnostic imaging”[Majr] OR “Polycystic Ovary Syndrome/epidemiology”[Majr] OR “Polycystic Ovary Syndrome/etiology”[Majr] OR ‘Polycystic Ovary Syndrome/genetics’[Majr] OR ‘Polycystic Ovary Syndrome/metabolism’[Majr] OR ‘Polycystic Ovary Syndrome/pathology’[Majr] OR ‘Polycystic Ovary Syndrome/physiopathology’[Majr] OR ‘Polycystic Ovary Syndrome/prevention and control’[Majr] ). Studies were selected based on the PICOS criteria (Chart 1).^(20)^ The primary outcomes analyzed were hyperandrogenism (measured by serum testosterone), insulin resistance, HDL and LDL cholesterol, total cholesterol, triglycerides, and BMI.

**Chart 1:** PICOS criteria used for study eligibility

PICOS criteria	Criteria used for eligibility
P	Women of reproductive age diagnosed with PCOS
I	Aerobic or resistance exercise
C	Comparison before and after aerobic or resistance exercise
O	BMI, testosterone, insulin, HDL, LDL, total cholesterol and triglycerides
S	Original articles

P: population; I: intervention; C: comparison; O: outcomes; S: study; PCOS: polycystic ovary syndrome; BMI: body mass index; HDL: high-density lipoprotein; LDL: low-density lipoprotein.

Only fully published articles involving women of reproductive age diagnosed with PCOS were included. All studies implemented a supervised aerobic or resistance exercise intervention, with a control group of participants who also had PCOS but did not receive any intervention. Inclusion and exclusion criteria are detailed in chart 2. Briefly, studies were eligible if they provided primary data (pre-intervention) and secondary data (post-intervention) and excluded if they involved dietary and drug interventions.

**Chart 2:** Eligibility criteria for inclusion and exclusion of research in this systematic review and meta-analysis

Inclusion criteria:	
1.	Original articles;
2.	Randomized controlled trials (RCTs) that compared a PCOS group with supervised aerobic or resistance exercise with a PCOS control group without intervention for meta-analytical analysis;
3.	Intervention lasting at least two weeks with a minimum exercise duration of 30 minutes a day;
4.	Women of reproductive age diagnosed with Polycystic Ovary Syndrome based on the NIH (1990) or Rotterdam ESHRE/ASRM (2003) criteria;
5.	Studies containing primary results of the intervention group and control group of BMI, testosterone, blood glucose, insulin, HDL, LDL, cholesterol and triglycerides and then the secondary results (after the intervention) of the same results;
6.	Studies published in English.
Exclusion criteria:	
1.	Systematic reviews, meta-analyses, literature reviews or abstracts;
2.	Studies involving only one high-intensity interval training (HIIT) group, with no other groups involving aerobic or resistance exercise compared to a control group;
3.	Study protocol and/or guidelines;
4.	Research involving any type of diet;
5.	Studies with pregnant women;
6.	Articles with no control group;
7.	Psychological/cognitive-behavioral analysis;
8.	Retrospective studies;
9.	Studies analyzing only medicines;
10.	Research that does not include aerobic or resistance exercise groups;
11.	Observational research;
12.	Case reports;
13.	Analyses of sexual function only;
14.	Research published in languages other than English;
15.	Animal model studies.

**Supplementary figure S1:**
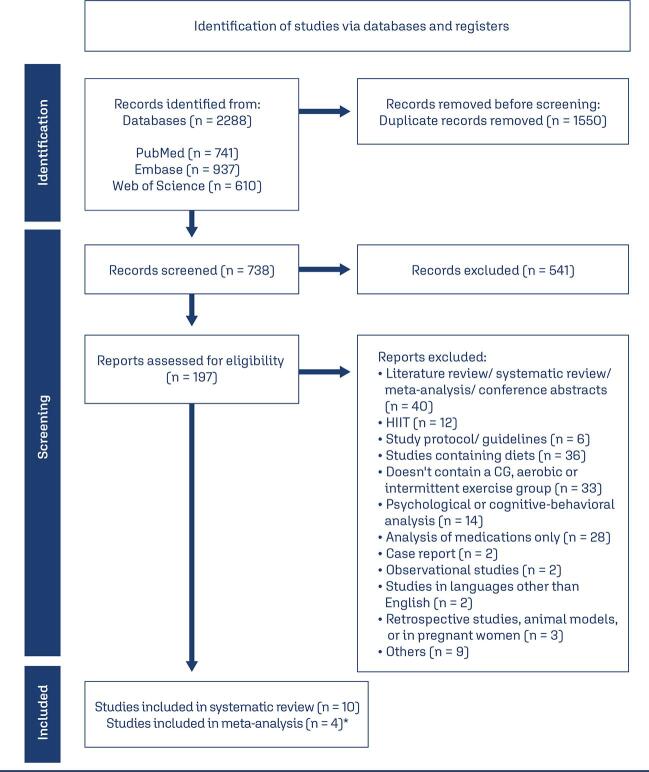
PRISMA Flow Diagram of the Study Selection Process for the Systematic Review and Meta-Analysis *One of the studies analyzed in the meta-analysis was divided into Group A and Group B, which correspond to a continuous aerobic exercise group and an intermittent aerobic exercise group, respectively.

**Supplementary figure S2:**
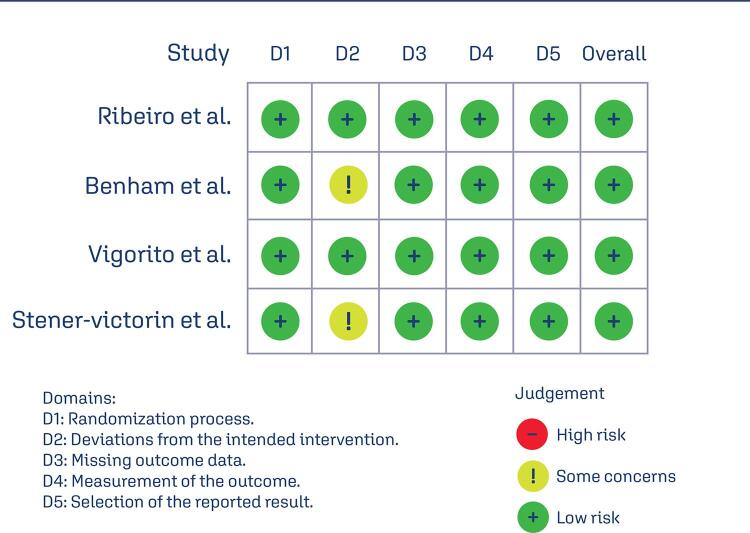
Risk of Bias Assessment and Funnel Plot for Publication Bias in Included Randomized Clinical Trials The meta-analysis of the five included studies showed positive SMD in the observed outcomes, ranging from 0.2185 to 0.2536, with all estimates being positive (100%). The pooled effect size, based on the random-effects model, was not statistically significant, with an average SMD of 0.2361 (95% CI: -0.0094 to 0.4815; p = 0.0594), indicating that the aerobic exercise intervention did not result in a significant change in HDL compared to the control (z = 1.8851). Heterogeneity across studies was minimal. The Q-test revealed no significant heterogeneity (Q(4) = 0.0084, p = 1.0000), and the tau^2^ estimate was 0.0000, with an *I*^2^ value of 0.0000%, indicating no observed variability in the true effects. These results suggest that the aerobic exercise intervention had consistent effects across the studies. Outlier analysis using studentized residuals found no studies with residuals larger than ±2.5758, indicating no presence of outliers. Similarly, Cook’s distance analysis did not identify any studies as overly influential. Lastly, both the rank correlation test and the regression test showed no evidence of funnel plot asymmetry, suggesting no indication of publication bias (rank correlation: p = 0.8167; regression: p = 0.9729)

The Rayyan Systematic Review web application, developed by the Qatar Computing Research Institute, organized and stored study data. After removing duplicates, two researchers (DCBC and MBC) independently reviewed the articles, and the studies approved by both reviewers were included. The screening process was conducted in three stages: title, abstract, and full-text review for eligible studies. Disagreements were resolved through discussion, and only studies with consensus were included. The PRISMA Flow Diagram (Supplementary figure S1) illustrates the process, while chart 3 summarizes the extracted data. The quality of RCTs was evaluated using the Cochrane risk-of-bias tool (RoB2), classifying studies as “high risk,” “low risk,” or “some concern” across five domains: randomization process, deviations from intended interventions, missing outcome data, outcome measurement, and selection of reported results. The quality assessment is presented in supplementary figure S2.

**Chart 3:** Summary of the studies included in the systematic review

First author (year)	Study Design	Participants and Distribution	Study Duration	Study Criteria and Participant Characteristics	Methods	Clinical and Metabolic Parameters
Aerobic Exercise						
Benham et al. (2021)^(21)^	RCT with 3 groups: CAET Control group HIIT group	47 participants: CAET group (n=14) Control group(n=17) HIIT group (n=16, not considered in the analysis)	26 weeks	Criteria: Rotterdam Age(y): 18 to 40 BMI (mean kg/m^2^): CAET group: 31,4 Control group: 31,1	CAET participants performed 40 minutes of moderate-intensity aerobic exercise (50% to 60% of HRR) using a Polar H10 HR sensor and Polar A370 watch	BMI, WC, SBP, DBP, VO2 max, Fasting Insulin, Fasting Glucose, HOMA2-IR, Total Cholesterol, HDL-C, LDL-C, TG, ALT, GGT.
Brown et al. (2009)^(22)^	RCT with 2 groups: Exercise group Control group	20 participants: Exercise group (n=8) Control group (n=12)	12 weeks	Criteria: NIH Age(y): 18 to 50 BMI (mean kg/m^2^): Exercise group: 37,9 Control group: 31,3	Moderate-intensity aerobic exercise on a treadmill (50% VO2 max) using a Polar Electro HR sensor	BMI, CC, SBP, DBP, FG score, TST, VO2 max, HOMA-IR, Glycemia, AUCglu, Insulin, AUCins, HbA1c, HDL, LDL, VLDL, TG.
Jedel et al. (2011)^(23)^	RCT with 3 groups: PCOS group Control group EA group	74 participants: Exercise group (n=30) Control group (n=15) EA group (n=29, not considered in the analysis)	16 weeks	Criteria: Rotterdam Age(y): 18 to 37 BMI (mean kg/m^2^): Exercise group: 27,7 Control group: 26,8	Regular aerobic exercise 3 days per week, including brisk walking or cycling, self-monitored with an ECG2 HR monitor to maintain a heart rate of ≥ 120 beats/min	BMI, VO2 max, TST, E1-S, E2, DHEA, 17G, SHBG, LH, FSH, FG score.
Lopes et al*.* (2018)^(24)^ Ribeiro et al. (2020)^(25)^ Ribeiro et al. (2021)^(26)^	RCT with 3 groups: CAT group IAT group Control group	87 participants: CAT group (n=28) IAT group (n=29) Control group (n=30)	16 weeks	Criteria: Rotterdam Age(y): 18 to 39 BMI (mean kg/m^2^): CAT group: 28,43 IAT group: 28,67 Control group: 29,09	Treadmill aerobic exercise three times per week, progressively increasing from 30min (week 1) to 50 min (week 16). Intensity levels: Light (50-64% HRmax), Moderate (64-77% HRmax), and Vigorous (77-94% HRmax)	BMI, WC, Hip Circumference, WHR, Body weight, Height, SBP, DBP, TST, A4, SHBG, FAI, E2, LH, FSH, Total Cholesterol, TG, HDL, LDL, Fasting Glycemia, Fasting Insulin, HOMA-IR, Homocysteine, CRP.
Stener-Victorin et al. (2009)^(27)^	RCT with 3 groups: Exercise group Control group EA group	20 participants: Exercise group (n=5) Control group (n=6) EA group (n=9, not considered in the analysis)	16 weeks	Criteria: Rotterdam Age(y) mean: Exercise group: 30,4 Control group: 31 BMI (mean kg/m^2^): Exercise group: 26,8 Control group: 28,0	Light to moderate-intensity aerobic exercise (bicycle ergometer, cycling, or brisk walking) for 30 to 45 minutes, 3 days per week.	BMI, WHR, SBP, DBP, HR, LH, FSH, FG score, Total TST, Free TST, SHBG, FAI, DHEA-S, Free T4, TSH, Glucose, Insulin, HOMA-IR, Cholesterol, TG, HDL, LDL.
Vigorito et al. (2007)^(28)^	RCT with 2 groups: Exercise group Control group	90 participants: Exercise group (n=45) Control group (n=45)	13 weeks	Criteria: Rotterdam Age(y) mean: 22 BMI (mean kg/m^2^): Exercise group: 29,3 Control group: 29,4	Aerobic exercise on a bicycle ergometer for 30 minutes at 60-70% of VO2 max, three times per week.	BMI, WC, WHR, FG score, Hemoglobin, FSH, LH, PRL, E2, P, 17-OHP, TST, A4, SHBG, FAI, DHEA-S, Fasting Glucose, Fasting Insulin, AUCglu, AUCins, AUCglu/AUCins ratio, Total Cholesterol, TG, HDL-C, LDL- C, CRP, VO2 max.
Resistance Exercise						
Almenning et al. (2015)^(29)^	RCT with 3 groups: Exercise group Control group HIIT group	31 participants: Exercise group (n=11) Control group (n=10) HIIT group (n=10, not considered in the analysis)	10 weeks	Criteria: Rotterdam Age(y) mean: 27,2 BMI (mean kg/m^2^): Exercise group: 27,4 Control group: 26,5	Resistance training involved eight dynamic strength exercises at 75% of 1RM, with 3 sets of 10 repetitions and one-minute rests between sets, gradually increasing the load. The control group was advised to engage in ≥ 150 minutes of exercise per week without follow-up during the 10-week intervention.	BMI, WC, VO2 max, Glucose, Insulin, HOMA-IR, TST, FAI, AMH, SHBG, DHEA-S, Total Cholesterol, HDL, LDL, TG, Homocysteine, Adiponectin, Leptin.
Vizza et al. (2016)^(30)^	RCT with 2 groups: Exercise group Control group	13 participants: 7 in Exercise group 6 in Control group	12 weeks	Criteria: Rotterdam Age(y): 18 to 42 BMI (mean kg/m^2^): 37,8	Resistance training was performed for 60 minutes on non-consecutive days, with 8 to 12 repetitions per set. Two sets of each exercise were completed in the first two weeks, progressing to three sets from week 3 onward.	BMI, WC, Hip Circumference, WHR, SBP, DBP, Fasting Insulin, Fasting Glucose, TST, SHBG, FAI, HbA1c.

1RM: One Repetition Maximum; 17G: 17β-Diol-17-Glucuronide; 17-OHP: 17 Alfa-Hydroxyprogesterone; A4: Androstenedione; Age (y): Age (years); ALT: Alanine Aminotransferase; AMH: Anti-Müllerian Hormone; AUCglu: Area Under The Curve Glucose; AUCins: Area Under The Curve Insulin; BMI: Body Mass Index; CAET: Continuous Aerobic Exercise Training; CAT: Continuous Aerobic Training; CRP: C-Reactive Protein; DBP: Diastolic Blood Pressure; DHEA: Dehydroepiandrosterone; DHEA-S: Dehydroepiandrosterone Sulfate; E1-S: E1 Sulfate; E2: Estradiol; EA: Electroacupuncture; FAI: Free Androgen Index; FG: Ferriman-Gallwey; FSH: Follicle Stimulating Hormone; GGT: Gamma-Glutamyl Transferase; HbA1c: Hemoglobin A1c; HDL: High Density Lipoproteins; HDL-C: High Density Lipoproteins Cholesterol; HOMA-IR: Homeostasis Model Assessment Of Insulin Resistance; HOMA2-IR: Homeostasis Model Assessment Index Of Insulin Resistance; HR: Heart Rate; HRmax: Heart Rate Maximum; IAT: Intermittent Aerobic Training; LDL: Low Density Lipoproteins; LDL-C: Low Density Lipoproteins Cholesterol; LH: Luteinizing Hormone; NIH: National Institutes Of Health; P: Progesterone; PRL: Prolactin; RCT: Randomized Controlled Trial; SBP: Systolic Blood Pressure; SHBG: Sex Hormone-Binding Globulin; T4: Thyroxine; TG: Triglycerides; TSH: Thyroid Stimulating Hormone; TST: Testosterone; VO2max: Maximal Oxygen Consumption; WC: Waist Circumference; WHR: Waist-to-Hip Ratio

The analysis used the standardized mean difference as the outcome measure, with a random-effects model fitted to the data. Heterogeneity (tau^2^) was estimated using the restricted maximum likelihood method,^(31)^ and Q tests^(32)^ and *I*^2^ statistics were reported. For tau^2^ > 0, a prediction interval was provided. Potential outliers and influential studies were identified using studentized residuals and Cook’s distances. Outliers were flagged based on a Bonferroni-corrected threshold (alpha = 0.05, two-tailed) for k studies, and studies with Cook’s distance exceeding the median plus six interquartile ranges were deemed influential. Funnel plot asymmetry was assessed via rank correlation and regression tests using standard errors as predictors.

## Results

A total of 2,288 articles were identified through PubMed (n = 741), Embase (n = 937), and Web of Science (n = 610). After removing 1,550 duplicates, 738 studies remained. Of these, 197 were identified as randomized controlled trials (RCTs) through the Rayyan website. After analyzing the titles and abstracts at the first screening level, 186 were further removed (Supplementary figure S1).

Of 197 randomized studies, 10 met the inclusion criteria. Eight involved aerobic exercise, with some distinguishing Continuous Aerobic Training (CAT) and Intermittent Aerobic Training (IAT). All studies included a control group. Seven studies used the Rotterdam Consensus criteria for PCOS diagnosis, and one used NIH criteria. Four aerobic exercise studies were included in the statistical analysis. Three articles included in the systematic review originated from the same study but presented distinct data analyses. Two of these included electroacupuncture (EA), aerobic exercise, and control groups; however, only data from the aerobic exercise and control groups were considered, with the EA group excluded from this analysis. Another study included HIIT, aerobic exercise, and a control group; the HIIT group was excluded. Two other studies focused solely on aerobic exercise and control groups.

Two studies addressed resistance exercises. One included HIIT, resistance exercise, and control groups; the HIIT group was excluded. The other study provided data at 3- and 6-month post-intervention; only the 3-month data, aligning with other studies, were used for statistical analysis, though the full 6-month data was considered in the systematic review.

The RCTs involving women with PCOS used aerobic exercises like cycling, walking, or treadmill running (Chart 4). The intervention periods in the exercise groups varied: three studies lasted 16 weeks,^(23-27)^ one lasted 12 weeks,^(22)^ another 3 months,^(28)^ and one 6 months.^(21)^ Exercise frequency was three sessions per week in seven studies,^(21,23-28)^ while one reported a mean 3.6 sessions per week.^(22)^ Exercise intensity was based on HRmax,^(24-26)^ VO2max,^(22,28)^ or 50 to 60% of HRR.^(21)^ Aerobic exercises included treadmill,^(22,24-26)^ stationary bike,^(28)^ or a combination of treadmill, walking, and cycling,^(23,27)^ or other similar aerobic activities.^(27)^

**Chart 4:** Overview of studies, exercise interventions, and outcomes following aerobic exercise in women with PCOS

Study (year)	Physical Intervention	Outcomes
Benham et al. (2021)^(21)^	CAT Control group	CAT: ↓ BMI; ↓WC. Control group: ↓ WC; ↑ insulin.
Brown et al. (2009)^(22)^	Exercise group Control group	Exercise group: ↑ VO2max; ↑ HDL; Improvement in insulin resistance (measured by AUCins); ↓ VLDL; ↓ TG Control group: There were no significant changes in the data.
Jedel et al. (2011)^(23)^	Exercise group Control group	Exercise group: ↓ circulating TST; ↓ E2; ↓ E1-S; ↓ 17G; ↑ VO2max; menstrual frequency increased Control group: Alteration in serum testosterone levels
Lopes et al*.* (2018)^(24)^ Ribeiro et al. (2020)^(25)^ Ribeiro et al. (2021)^(26)^	CAT IAT Control group	CAT: ↓ Total cholesterol; ↓ WC; ↓ Hip circumference; ↓ LDL; ↓ TST; ↓ cardiac output. IAT: ↓WC; ↓ WHR; ↓ FAI; ↓TST. Control group: ↑ WC; ↑ body fat; ↓ total cholesterol; ↓ LDL.
Stener-Victorin et al. (2009)^(27)^	Exercise group Control group	Exercise group: ↓ body weight; ↓ BMI; ↓ insulin; ↑ SHBG; ↑ FAI. Control group: ↓ LH; ↓ FSH; ↓ insulin.
Vigorito et al. (2007)^(28)^	Exercise group Control group	Exercise group: Improvement in VO_2_max and AUCins; ↓ BMI; ↓ WC; ↓ WHR; ↓ CRP; ↓ fasting insulin; ↑ AUCglu/AUCins ratio Control group: ↑ CRP

↑ indicates gain or increase; ↓ Indicates reduction; AUC: area under curve; AUCglu: glucose area under curve; AUCins insulin area under curve; AUCglu/AUCins: AUC glucose/AUC insulin ratio; BMI: body mass index; CAT: continuous aerobic training; CG: control group; CRP: C-reactive protein; E1-S: E1 sulfate; E2: estradiol; FAI: free androgen index; FSH: follicle-stimulating hormone; HDL: High Density Lipoprotein; IAT: intermittent aerobic training; LDL: low-density lipoprotein; LH: luteinizing hormone; SHBG: sex hormone-binding globulin; VLDL: very low-density lipoprotein; VO_2_ max: maximal oxygen consumption; 17G: 17β-diol-17-glucuronide; WC: waist circumference; WHR: waist-to-hip ratio

Aerobic exercise reduced BMI (-4.5%, p < 0.05), WC (p < 0.01), WHR (p < 0.05), and CRP levels (-10%, p < 0.001) in Vigorito et al.^(28)^After 16 weeks, the exercise group showed reductions in body weight (p = 0.004) and BMI (p = 0.004) compared to the control group, but no WHR differences.^(27)^ Benham et al.^(21)^ found significant BMI reduction (-1.0 kg/m^2^, p = 0.01) after six months, with WC reductions both the CAT (-6.9 cm) and control group (-4.5 cm). CAT and IAT groups showed significant WC (p = 0.045 and p=0.014, respectively) and WHR (p = 0.032 and p=0.012, respectively) decreases, while WC increased in the control group (p = 0.049).^(24-26)^ Other studies reported no significant BMI changes.^(23-26)^

In a three-month intervention, cycling for 30 minutes, three times a week at 60–70% of VO2max improved fasting insulin levels (p < 0.01), AUCins (p < 0.001), and VO2max compared to the control group. Additionally, in the intervention group, the ratio of AUCgli/AUCins increased (p < 0.001), with significant differences (p < 0.001) between the PCOS-T group and the control group over three months, compared to baseline. No significant changes in fasting glucose levels were reported.^(28)^ After 16 weeks of aerobic exercise, three times a week for 30–45 minutes, fasting insulin levels decreased from 8.0 ± 4.5 mU/l to 6.4 ± 2.8 mU/l in the intervention group. The control group also showed a decrease in fasting insulin levels from 9.2 ± 6.7 mU/l to 7.8 ± 3.1 mU/l, with no significant changes in blood glucose levels.^(27)^

In the study by Brown et al. (2009),^(22)^ no significant changes in insulin sensitivity were observed after training, including insulin sensitivity measured by the intravenous glucose tolerance test (IVGTT) with minimal model analysis. However, the intervention group demonstrated an improvement in insulin resistance as measured by AUCins compared to the control group (-4,930.7 vs +476.5; p = 0.083). Benham et al. (2021)^(21)^ reported an increase in fasting insulin levels in the control group by 19.5 mUI/L (95% CI 0.9–38.2; p = 0.04), while no changes were observed in the CAT group. Fasting glucose levels remained unchanged in both groups. No significant changes in fasting insulin or glucose levels were observed in other studies.^(24-26)^

Total cholesterol (p ≤ 0.001) and LDL (p = 0.030) levels decreased after CAT program. However, there was also a reduction in total cholesterol (p= 0.010) in the control group, which did not receive any intervention. No significant changes were observed in HDL or triglyceride levels.^(24-26)^Brown et al. (2009)^(22)^ investigates the effects of exercise on lipoprotein particles in women with PCOS and observed that the intervention group showed a decrease in calculated triglycerides (−44.3 vs +10.6 mg·dL^1^; *P* = 0.003) and VLDL triglycerides (−41.6 vs +11.6 mg·dL^1^; *P* = 0.003) after 12 weeks of aerobic exercise, compared to the control group. No significant differences in total cholesterol, HDL, LDL, or triglyceride levels were observed in other studies included in the analysis.^(21,27,28)^

A 16-week RCT comparing IAT and CAT on a treadmill showed a reduction in total testosterone levels in both groups: CAT (p ≤ 0.001) and IAT (p = 0.019). No significant changes were found in androstenedione, FSH, LH, estradiol, or SHBG levels. ^(24-26)^ In another study, the PCOS-T group showed a significant reduction in testosterone levels (-0.04 ± 0.14 ng/ml) after 16 weeks compared to the control group (0.01 ± 0.09 ng/ml), with no changes in FSH, LH, DHEA, or SHBG levels.^(23)^Stener-Victorin et al. (2009)^(27)^reported an increase in SHBG levels (31.6 ± 9.7 to 54.6 ± 53.9) in the aerobic exercise group after 16-week of training, but no changes in testosterone, FSH, or LH levels. Interestingly, the control group without supervised training experienced a decrease in FSH (4.8 ± 1.0 to 3.9 ± 1.3) and LH (10.3 ± 11.0 to 5.8 ± 2.2). In the study by Vigorito et al. (2007),^(28)^ no significant changes were observed in testosterone, progesterone, FSH, LH, SHBG, prolactin, estradiol, androstenedione, FAI, and DHEA-S levels.

The RCTs involving resistance exercises for women with PCOS included exercises such as squats, calf raises, triceps extensions, leg curls, lateral pulls, seated rows, leg press, bench press, bicep curls, and abdominal curls. Participants were also encouraged to practice calisthenics at home on non-training days, including wall squats, oblique curls, lateral leg raises, core stabilization exercises, knee push-ups, and hip external rotations (‘clamshells’).^(30)^Another study employed eight dynamic strength exercises at 75% of one repetition maximum (1RM).^(29)^The details of these studies are summarized in chart 5.

**Chart 5:** Outcomes of resistance exercise interventions in included studies

Study	Outcomes
Almenning et al. (2015)^(29)^	Exercise group: ↓ Fat percentage; ↓FAI; ↑ fat-free mass; ↓ Anti-Müllarian Hormone Control group: There were no significant changes in this group
Vizza et al. (2016)^(30)^	- Exercise group: ↑ weight (resulting from muscle hypertrophy); ↑ BMI; ↑ lean mass; ↑ fat-free mass; ↑ fasting glucose; ↓ WC; ↓ HbA1c Control group: There were no significant changes in this group.

↑ indicates gain or increase; ↓ Indicates reduction. AMH: Anti-Müllarian hormone; BMI: body mass index; FAI: free androgen index; HbA1c: hemoglobin A1c; WC: waist circumference

Two articles were included in the analysis of resistance exercise. The first study explored the feasibility of progressive resistance training over 12 weeks, with sessions held on non-consecutive days. The experimental group (PCOS-T) consisted of women with PCOS who participated in 60-minute resistance training sessions. Each exercise was performed until neuromuscular fatigue, with 8 to 12 repetitions per set, and the load was progressively increased as participants’ strength improved. During the first two weeks, participants completed two sets per exercise, increasing to three sets from week 3 onward. In contrast, the control group did not receive any intervention and was instructed to maintain their usual lifestyle.^(30)^

The second study examined the effects of HIIT and resistance training on metabolic, cardiovascular, and hormonal outcomes in women with PCOS. Three groups were analyzed: the HIIT group, the PCOS group engaged in resistance training (PCOS-T), and a control group.^(29)^ For this systematic review, only data from the PCOS group practicing resistance exercise and the control group were considered.

In the study conducted by Almenning et al. (2015),^(29)^ the intervention lasted 10 weeks and involved the PCOS-T group performing eight dynamic strength exercises at 75% of their 1RM. Each session consisted of three sets of 10 repetitions per exercise, with a one-minute rest between sets. The load was progressively increased as participants’ strength improved. In the control group, participants were advised to engage in ≥150 minutes of exercise per week without any supervision during the 10-week intervention period.

In the study by Vizza et al. (2016)^(30)^an increase in body weight (p = 0.01) and BMI (p = 0.04) was observed in the PCOS women compared to the control group. Additionally, there was a reduction in WC (p = 0.03), along with an increase in lean mass (p = 0.01) and fat-free mass (p = 0.005). No changes were observed in body fat percentages or fat mass between the PCOS-T and control groups. In contrast, Almenning et al. (2015),^(29)^ did not observe changes in body weight or WC after intervention in the studied groups. However, a reduction in body fat percentage (-1.6; 95% CI: -2.5, -0.7) and an increase in fat-free mass (1.2; 95% CI: 0.4, 2.1) were observed in PCOS group after resistance training.

Throughout the intervention, a decrease in HbA1c levels was observed in the PCOS-trained women compared to the control group (p = 0.03). However, an unexpected significant increase in fasting blood glucose levels was found within the experimental group (p= 0.03)^(30)^. In contrast, fasting glucose and insulin did change in the study by Almenning et al. (2015).^(29)^

In Almenning et al. (2015),^(29)^ the trained PCOS group showed a decrease in the FAI (-0.7, 95% CI: -1.3, -0.1), with no significant changes in testosterone, DHEAS, or SHBG levels. Similarly, Vizza et al. (2016)^(30)^ did not show changes in testosterone or SHBG levels or FAI.

**Supplementary figure S3:**
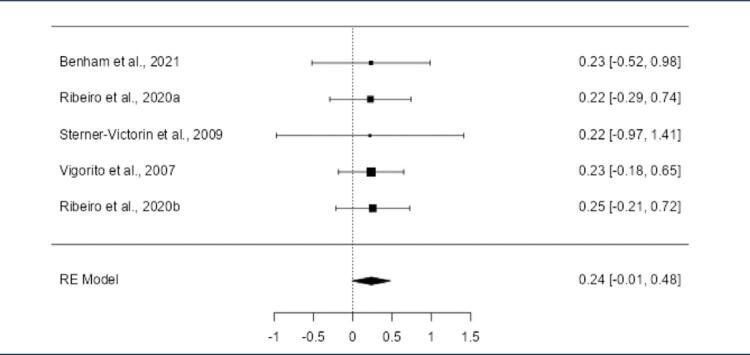
Forest plot of the meta-analysis evaluating the effects of aerobic exercise on HDL levels The meta-analysis of the five included studies revealed positive SMD in the observed outcomes, ranging from 0.2128 to 0.2242, with all estimates being positive (100%). The pooled effect size, based on the random-effects model, was not statistically significant, with an average SMD of 0.2205 (95% CI: -0.0249 to 0.4658; p = 0.0782), indicating that aerobic exercise did not result in a significant change in LDL levels compared to the control group (z = 1.7612). Heterogeneity across studies was minimal. The Q-test revealed no significant heterogeneity (Q(4) = 0.0009, p = 1.0000), and the tau^2^ estimate was 0.0000, with an *I*^2^ value of 0.0000%, indicating no variability in the true effects. These findings suggest that the effects of aerobic exercise on LDL levels were consistent throughout the studies. Outlier analysis using studentized residuals showed no studies with residuals larger than ±2.5758, indicating no outliers. Similarly, Cook›s distance analysis did not identify any studies as overly influential. Finally, both the rank correlation test and the regression test showed no evidence of funnel plot asymmetry, suggesting no publication bias (rank correlation: p = 0.8167; regression: p = 0.9954)

The meta-analysis focused on aerobic exercise due to limited data on resistance exercise. Four studies included: Benham et al. (2021),^(21)^ Ribeiro et al. (2020),^(25)^ Stener-Victorin et al. (2009),^(27)^ and Vigorito et al. (2007).^(28)^Supplementary table 1 summarizes the outcomes for both PCOS and control groups. The pooled effect size showed no statistical significance for HDL (Supplementary figure S3), LDL (S4), testosterone (S5), and BMI (S6). Significant effects were observed for insulin, cholesterol, and triglycerides.

**Supplementary table 1:** Changes in anthropometric, metabolic, and hormonal parameters in trained and control groups before and after the intervention period

Study	Year	Study duration (weeks)	Outcome	Control Group*					Aerobic Training				
					Before		After			Before		After	
				n	Mean	SD	Mean	SD	n	Mean	SD	Mean	SD
Benham et al.^(21)^	2021	13	BMI (Kg/m^2^)	17	31,1	2,2	31,1	2,2	14,0	31,4	2,6	31,0	2,5
Ribeiro et al.^(25)^	2020a	16	BMI (Kg/m^2^)	30	29,1	5,2	29,3	5,4	28,0	28,4	5,6	28,2	5,7
Stener-Victorin et al.^(27)^	2009	16	BMI (Kg/m^2^)	6	28,0	6,2	28,5	6,2	5,0	26,8	4,8	26,4	4,8
Vigorito et al.^(28)^	2007	13	BMI (Kg/m^2^)	45	29,4	3,5	29,3	3,2	45,0	29,3	2,9	28,0	2,9
Ribeiro et al.^(25)^	2020b	16	BMI (Kg/m^2^)	45	29,4	3,5	29,3	3,2	29	28,7	4,8	28,5	4,8
Benham et al.^(21)^	2021	13	No data										
Ribeiro et al.^(25)^	2020a	16	Testosterone (ng/dl)	30	86,0	37,0	100,0	46,0	28,0	117,0	50,0	93,0	38,0
Stener-Victorin et al.^(27)^	2009	16	Testosterone (ng/dl)	6	60,5	23,0	57,6	20,1	5,0	57,6	25,9	57,6	8,6
Vigorito et al.^(28)^	2007	13	Testosterone (ng/dl)	45	72,0	14,4	69,1	11,5	45,0	66,2	20,1	60,5	17,2
Ribeiro et al.^(25)^	2020b	16	Testosterone (ng/dl)	45	29,4	3,5	29,3	3,2	29	108	52	88	54
Benham et al.^(21)^	2021	13	Insulin (mIU/L)	17	99,5	15,4	114,0	17,6	14,0	83,4	17,8	86,4	19,8
Ribeiro et al.^(25)^	2020a	16	Insulin (mIU/L)	30	12,8	8,5	12,4	9,8	28,0	11,3	8,1	11,2	8,3
Stener-Victorin et al.^(27)^	2009	16	Insulin (mIU/L)	6	9,2	6,7	7,8	3,1	5,0	8,0	4,5	6,4	2,8
Vigorito et al.^(28)^	2007	13	Insulin (mIU/L)	45	20,2	3,8	20,4	3,6	45,0	20,1	3,5	18,3	3,0
Ribeiro et al.^(25)^	2020b	16	Insulin (mIU/L)	45	29,4	3,5	29,3	3,2	29	9,5	7,2	10,4	7
Benham et al.^(21)^	2021	13	HDL (mg/dL)	17	50,3	3,9	46,4	3,9	14,0	42,5	3,9	42,5	3,9
Ribeiro et al.^(25)^	2020a	16	HDL (mg/dL)	30	50,0	13,0	48,0	13,0	28,0	46,0	9,0	44,0	10,0
Stener-Victorin et al.^(27)^	2009	16	HDL (mg/dL)	6	65,7	11,6	58,0	15,5	5,0	54,1	11,6	54,1	7,7
Vigorito et al.^(28)^	2007	13	HDL (mg/dL)	45	59,0	19,0	58,0	16,0	45,0	53,8	11,6	55,2	12,9
Ribeiro et al.^(25)^	2020b	16	HDL (mg/dL)	45	29,4	3,5	29,3	3,2	29	49	11	47	10
Benham et al.^(21)^	2021	13	LDL (mg/dL)	17	100,5	7,7	112,1	7,7	14,0	96,7	7,7	100,5	7,7
Ribeiro et al.^(25)^	2020a	16	LDL (mg/dL)	30	116,0	32,0	108,0	27,0	28,0	112,0	24,0	102,0	23,0
Stener-Victorin et al.^(27)^	2009	16	LDL (mg/dL)	6	85,1	11,6	85,1	19,3	5,0	88,9	19,3	85,1	23,2
Vigorito et al.^(28)^	2007	13	LDL (mg/dL)	45	72,5	21,6	75,9	24,9	45,0	76,3	18,3	73,2	23,8
Ribeiro et al.^(25)^	2020b	16	LDL (mg/dL)	45	29,4	3,5	29,3	3,2	29	112	23	106	23
Benham et al.^(21)^	2021	13	Cholesterol (mg/dL)	17	174,0	7,7	181,8	7,7	14,0	158,6	7,7	162,4	7,7
Ribeiro et al.^(25)^	2020a	16	Cholesterol (mg/dL)	30	188,0	34,0	178,0	24,0	28,0	185,0	30,0	171,0	28,0
Stener-Victorin et al.^(27)^	2009	16	Cholesterol (mg/dL)	6	158,5	11,6	154,7	27,1	5,0	154,7	23,2	154,7	19,3
Vigorito et al.^(28)^	2007	13	Cholesterol (mg/dL)	45	154,0	16,0	156,0	17,0	45,0	153,0	18,1	151,0	16,4
Ribeiro et al.^(25)^	2020b	16	Cholesterol (mg/dL)	45	29,4	3,5	29,3	3,2	29	179	29	174	27
Benham et al.^(21)^	2021	13	Triglycerides (mg/dL)	17	115,2	17,7	115,2	17,7	14,0	97,4	17,7	88,6	17,7
Ribeiro et al.^(25)^	2020a	16	Triglycerides (mg/dL)	30	112,0	56,0	103,0	59,0	28,0	151,0	172,0	144,0	139,0
Stener-Victorin et al.^(27)^	2009	16	Triglycerides (mg/dL)	6	62,0	17,7	70,9	35,4	5,0	62,0	26,6	62,0	8,9
Vigorito et al.^(28)^	2007	13	Triglycerides (mg/dL)	45	112,0	21,0	111,0	23,0	45,0	114,6	20,3	113,3	23,3
Ribeiro et al.^(25)^	2020b	16	Triglycerides (mg/dL)	45	29,4	3,5	29,3	3,2	29	99	54	107	61

*The control group consisted of women with PCOS who did not engage in physical activity. Ribeiro et al. (2020a):^(25)^ continuous aerobic training; Ribeiro et al. (2020b):^(25)^ intermittent aerobic training; BMI: body mass index; SD: standard deviation; HDL: High Density Lipoprotein; LDL: low-density lipoprotein

**Figure 1:**
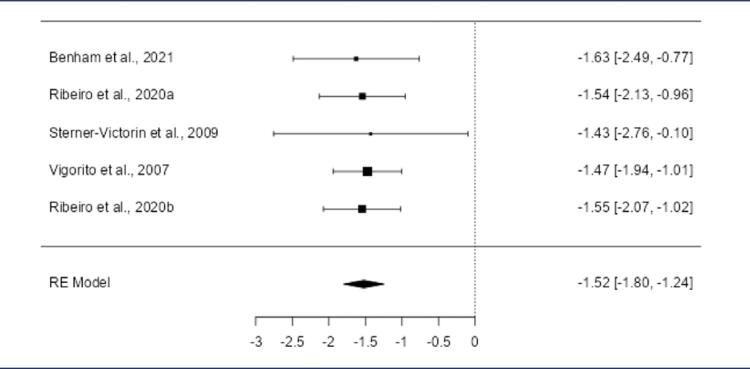
Forest plot of the meta-analysis evaluating the effects of aerobic exercise on insulin levels The pooled standardized mean difference (SMD) was -1.5223 (95% CI: -1.7996 to -1.2449; p < 0.0001), indicating a significant reduction in insulin levels following aerobic exercise compared to the control group. Heterogeneity across studies was negligible (Q(4) = 0.1315, p = 0.9979; I^2^ = 0.0000%), and no outliers or publication bias were detected

The meta-analysis revealed a negative standardized mean difference (SMD) across studies for insulin levels (-1.5223, 95% CI: -1.7996 to -1.2449; p < 0.0001) (Figure 1). Although the number of included studies was small, the selected studies demonstrated homogeneity (Q(4) = 0.1315, p = 0.9979, *I*^2^ = 0. 0000%). Outlier and Cook’s distance analyses identified no influential studies. Similarly, Cook›s distance analysis did not identify any studies as overly influential in affecting the model. Funnel plot tests showed no publication bias (rank correlation: p = 0.8167; regression: p = 0.9511).

**Figure 2:**
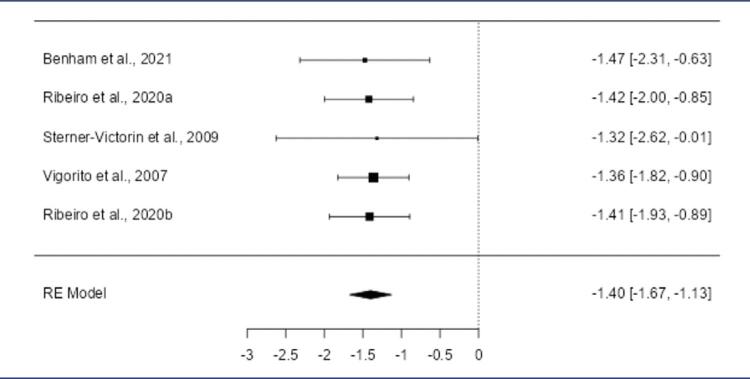
Forest plot of the meta-analysis evaluating the effects of aerobic exercise on cholesterol levels The pooled SMD was -1.3989 (95% CI: -1.6714 to -1.1264; p < 0.0001), demonstrating a significant reduction in cholesterol levels following aerobic exercise compared to the control group. Minimal heterogeneity was observed (Q(4) = 0.0773, p = 0.9993; I^2^ = 0.0000%). One study (Vigorito et al., 2007^(28)^ was identified as overly influential, but the model remained robust. No evidence of publication bias was observed.

A reduction in total cholesterol levels was observed between PCOS and controls group since the pooled SMD was negative (-1.3989; 95% CI: -1.6714 to -1.1264; p < 0.0001) (Figure 2). The aerobic exercise intervention was consistent across the studies since the heterogeneity was negligible (Q(4) = 0.0773, p = 0.999; I^2^ = 0.0%). One study^(28)^ was flagged as influential by Cook’s distance analysis, but the model remained robust. Publication bias was unlikely (rank correlation: p = 0.483; regression: p = 0.977).

**Figure 3:**
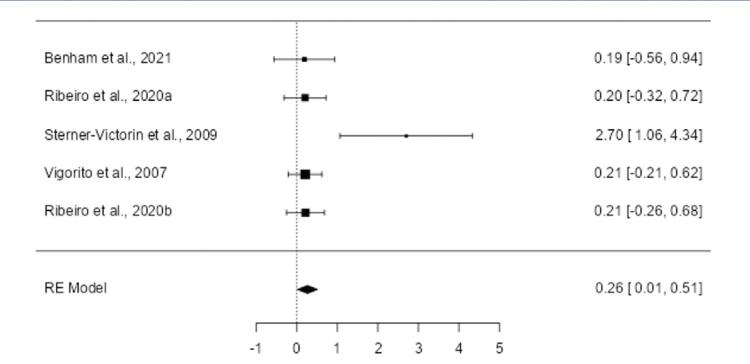
Forest plot of the meta-analysis evaluating the effect of aerobic exercise on triglyceride levels The pooled SMD was 0.2614 (95% CI: 0.0136 to 0.5091; p = 0.0387), indicating a significant reduction in triglyceride levels with aerobic exercise compared to the control group. Heterogeneity was minimal (Q(4) = 8.7300, p = 0.0682; I^2^ = 0.0014%). One potential outlier (Sterner-Victorin et al., 2009) was identified, and mixed evidence of publication bias was observed (regression test: p = 0.0112; rank correlation: p = 0.0833)

The pooled SMD was positive (0.2614; 95% CI: 0.0136 to 0.5091; p = 0.039) (Figure 3), reflecting reduced triglycerides. While the Q-test for heterogeneity was not statistically significant, some degree of heterogeneity may still be present in the true outcomes (Q(4) = 8.73, p = 0.068; I^2^ = 0.1%). The 95% prediction interval for the true outcomes was 0.0136 to 0.5091, showing a slight heterogeneity, the true effects across studies are generally consistent with the estimated average outcome. One study^(27)^ was identified as a potential outlier, but no overly influential studies were found. Mixed evidence of publication bias was observed (rank correlation: p = 0.083; regression: p = 0.011).

## Discussion

This systematic review and meta-analysis evaluated exercise interventions in women with PCOS, focusing primarily on aerobic exercise due to limited data on resistance training. A total of 10 studies with 382 participants (318 in the meta-analysis) were included. Interventions averaged 12.1 weeks (10–26 weeks). Aerobic exercise studies involved 11–90 participants, while only one study met the criteria for resistance training, exposing a literature gap. Most studies were homogeneous regarding analyzed variables.

PCOS is associated with obesity, infertility, and metabolic dysfunction.^(2)^ Intervention groups showed reductions in BMI, WC, and WHR, along with improved metabolic and hormonal markers, such as testosterone and insulin, which enhance reproductive function and quality of life.^(33,34)^ A 5% weight loss can improve ovulation and reduce metabolic complications.^(35)^ Thus, regular exercise may be considered a viable treatment for women with PCOS, particularly those seeking conception.^(36,37)^Maintaining BMI within a healthy range reduces the risks of gestational complications, including hypertensive disorders and gestational diabetes.^(38)^

Visceral adiposity links insulin resistance and hyperandrogenism in PCOS,^(39-43)^ although non-obese women with PCOS also experience insulin resistance,^(44)^ contributing to hyperandrogenism.^(45,46)^ Lifestyle changes, including exercise, reduce insulin resistance more effectively than metformin alone.^(47)^ A Mendelian randomization study linked visceral adipose tissue mass to higher PCOS risk (OR [95% CI] = 1.15 [1.08 – 1.23], p = 3.24 x 10^-5^), and dysfunctions like preeclampsia.^(34)^

Women engaging in vigorous exercise exhibited lower BMI, HOMA-IR, and metabolic syndrome indices, and higher HDL and SHBG levels. Additionally, it reduces metabolic syndrome risk by 22% per hour (OR = 0.78 [0.62–0.99]).^(48)^ Supervised activity improved androstenedione and SHBG levels.^(49)^ Regular physical exercise and weight loss not only improves glucose tolerance,^(50)^ ovulatory function, and menstrual cycles.^(51,52)^ Resistance training benefits women with PCOS, including muscle hypertrophy, cardiometabolic outcomes,^(30)^ and reductions in BMI and body fat percentage, along with improved testosterone levels.^(29)^ A study by Rao et al. (2022)^(53)^ analyzed the effects of HIIT and resistance training on testosterone levels and anthropometric measurements in women with PCOS, reporting significant improvements in BMI, body fat percentage, and serum testosterone levels after 12 weeks. Similarly, Ennour-Idrissi et al. (2015),^(49)^ observed significant effects on free testosterone levels from resistance and high-intensity exercises.

Turan et al. (2015)^(54)^ reported improvements in both anthropometric and cardiovascular parameters in eutrophic women after 8 weeks of structured aerobic and resistance exercise. The intervention resulted in reductions in WC, waist-to-height ratio, diastolic blood pressure, and respiratory rate, along with a significant increase in VO2max. Aerobic exercise enhances oxygen uptake, promotes the oxidation of free fatty acids, and utilizes glucose as an energy source.^(55)^ This elevation in aerobic metabolism reduces body fat and improves cardiorespiratory capacity.^(56,57)^ Low cardiorespiratory fitness increases cardiovascular disease risk and hinders physical activity and daily task engagement.^(58,59)^

Roessler et al. (2013)^(60)^ investigated the effects of 16 weeks of high-intensity aerobic exercise on body composition and VO2max in overweight women with PCOS, finding reductions in waist circumference and BMI, along with an increase in VO2max. Similarly, Wu et al. (2021)^(61)^ evaluated the effects of a 12-week stationary bike exercise program on AMH levels and oxidative stress in women with PCOS, finding reductions in BMI and increases in VO2max. A systematic review and meta-analysis concluded that vigorous-intensity exercise positively impacts body composition, insulin resistance, and cardiorespiratory fitness in women with PCOS.^(62)^

Among the included studies, VO2max levels increased following aerobic exercise interventions.^(22,23,28)^ In contrast, studies examining resistance exercise either did not report VO2max data,^(30)^ or showed no significant changes.^(29)^A systematic review and meta-analysis found increases in VO2max for both aerobic and resistance exercise groups; however, resistance training was less effective than aerobic training in improving VO2max.^(63)^ Enhancing VO2max is crucial for improving cardiorespiratory fitness, particularly for patients with PCOS, though research on its significance in this population remains limited.^(59)^ While the optimal type and intensity of resistance exercises for managing remain unclear, their potential impact warrants further investigation.^(64)^

These findings underscore the importance of resistance exercise in improving anthropometric, cardiometabolic, and hormonal outcomes in women with PCOS. Aerobic exercise improves anthropometric, metabolic, hormonal, and reproductive parameters in women with PCOS, highlighting its role in managing the syndrome’s clinical features. The reduction in obesity, evidenced by decreases in BMI, WC, WHR and waist-to-height ratio, underscores the importance of maintaining these indices for effective weight management. Additionally, aerobic exercise improves insulin levels, reducing hyperinsulinemia and insulin resistance risks. The reduction in serum testosterone levels following structured exercise program highlights their positive impact on hormonal regulation, alleviating hyperandrogenism-related clinical manifestations. Therefore, aerobic exercise is highly effective in improving outcomes in patients with PCOS, mitigating the syndrome’s adverse effects.

The strengths of this study lie in its comprehensive bibliographic research and clearly defined eligibility criteria. However, limitations included a limited number of RCTs meeting eligibility criteria and variations in exercise protocols across studies. These variations may have contributed to the heterogeneity in some outcomes. Additionally, not all studies employing resistance exercise reported usable data for the meta-analysis, preventing adequate comparisons and highlighting a research gap. Given the promising potential of exercise interventions, there is an urgent need for further investigations and RCTs focusing on structured exercise programs for PCOS patients, aiming to yield statistically significant results.

## Conclusion

This systematic review analyzed 10 randomized controlled trials and 2 studies focusing on supervised aerobic and resistance exercise interventions in women with PCOS. The findings underscore the beneficial effects of these exercise programs on a range of PCOS-related outcomes, including significant improvements in anthropometric measurements, metabolic profiles, hormonal balance, and reproductive health. These results highlight the importance of incorporating structured exercise interventions as a viable therapeutic strategy for managing PCOS and enhancing overall well-being in affected women.

**Supplementary figure S4.**
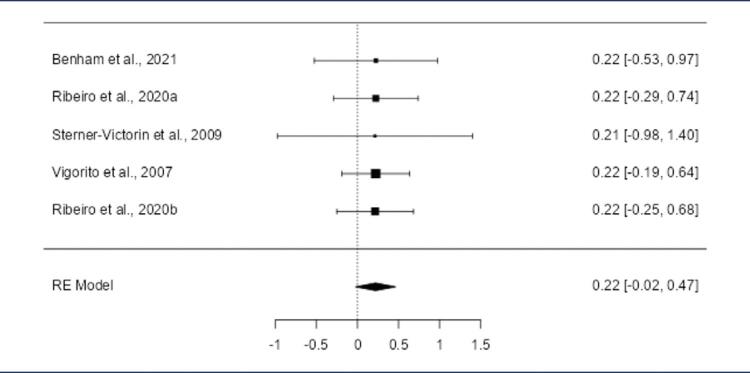
Forest plot of the meta-analysis evaluating the effect of aerobic exercise on LDL levels The meta-analysis of the five included studies revealed positive SMD in the observed outcomes, ranging from 0.2128 to 0.2242, with all estimates being positive (100%). The pooled effect size, based on the random-effects model, was not statistically significant, with an average SMD of 0.2205 (95% CI: -0.0249 to 0.4658; p = 0.0782), indicating that aerobic exercise did not result in a significant change in LDL levels compared to the control group (z = 1.7612). Heterogeneity across studies was minimal. The Q-test revealed no significant heterogeneity (Q(4) = 0.0009, p = 1.0000), and the tau² estimate was 0.0000, with an I² value of 0.0000%, indicating no variability in the true effects. These findings suggest that the effects of aerobic exercise on LDL levels were consistent throughout the studies. Outlier analysis using studentized residuals showed no studies with residuals larger than ±2.5758, indicating no outliers. Similarly, Cook›s distance analysis did not identify any studies as overly influential. Finally, both the rank correlation test and the regression test showed no evidence of funnel plot asymmetry, suggesting no publication bias (rank correlation: p = 0.8167; regression: p = 0.9954)

**Supplementary figure S5:**
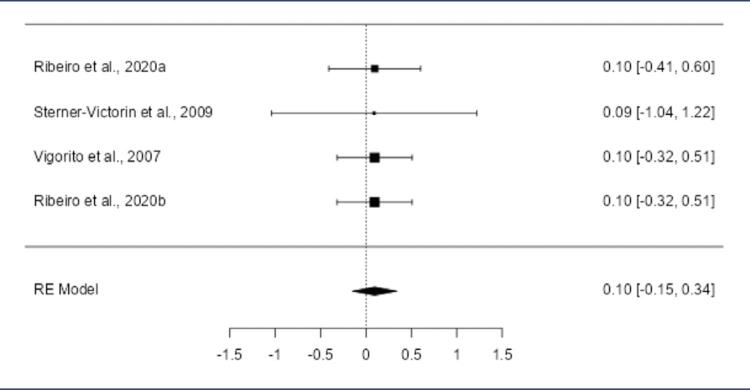
Forest plot of the meta-analysis evaluating the effect of aerobic exercise on testosterone levels. The meta-analysis of the four included studies revealed positive SMD in the observed outcomes, ranging from 0.0896 to 0.0965, with all estimates being positive (100%). The pooled effect size, based on the random-effects model, was not statistically significant, with an average SMD of 0.0954 (95% CI: -0.1517 to 0.3425; p = 0.4492). This indicates that aerobic exercise did not lead to a significant change in testosterone levels compared to the control group (z = 0.7568). The Q-test indicated no significant heterogeneity among the studies (Q(3) = 0.0001, p = 1.0000), and the tau^2^ estimate was 0.0000, with an *I*^2^ value of 0.0000%, suggesting no variability in the true effects. An examination of the studentized residuals found no studies with residuals larger than ±2.4977, indicating no presence of outliers. Similarly, Cook’s distance analysis did not identify any studies as overly influential. Furthermore, both the rank correlation test and the regression test showed no evidence of funnel plot asymmetry, suggesting no publication bias (rank correlation: p = 1.0000; regression: p = 0.9928).

**Supplementary figure S6:**
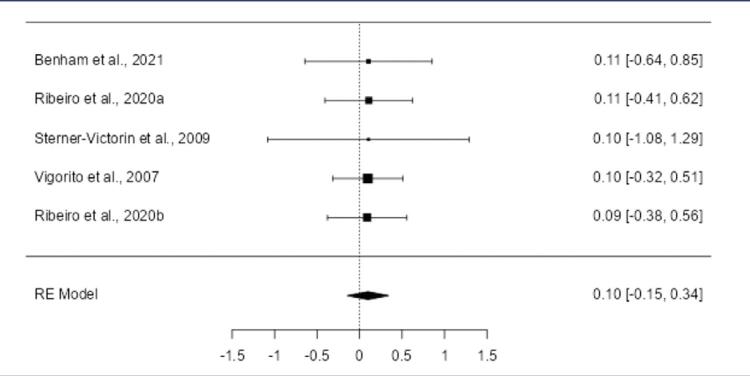
Forest plot of the meta-analysis evaluating the effect of aerobic exercise on BMI The meta-analysis of the five included studies revealed positive SMD in the observed outcomes, ranging from 0.0893 to 0.1088, with all estimates being positive (100%). The pooled effect size, based on the random-effects model, was not statistically significant, with an average SMD of 0.0987 (95% CI: -0.1461 to 0.3435; p = 0.4293, Figure 4). This indicates that aerobic exercise did not lead to a significant change in BMI compared to the control group (z = 0.7904). The Q-test indicated no significant heterogeneity among the studies (Q(4) = 0.0035, p = 1.0000), and the tau^2^ estimate was 0.0000, with an I^2^ value of 0.0000%, suggesting no variability in the true effects. Outlier analysis using studentized residuals found no studies with residuals larger than ±2.5758, indicating no presence of outliers. Similarly, Cook’s distance analysis did not identify any studies as overly influential. Finally, both the rank correlation test and the regression test showed no evidence of funnel plot asymmetry, suggesting no publication bias (rank correlation: p = 0.8167; regression: p = 0.9804).
